# Impact of metformin on the risk and treatment outcomes of tuberculosis in diabetics: a systematic review

**DOI:** 10.1186/s12879-019-4548-4

**Published:** 2019-10-17

**Authors:** Xinyu Yu, Ling Li, Liangtao Xia, Xin Feng, Fan Chen, Shiyi Cao, Xiang Wei

**Affiliations:** 1 Division of Cardiothoracic and Vascular Surgery, Tongji Hospital, Tongji Medical College, Huazhong University of Science and Technology, No. 1095 Jiefang Avenue, Wuhan, 430030 China; 2 Department of Infectious Diseases, Institute of Infection and Immunology, Union Hospital, Tongji Medical College, Huazhong University of Science and Technology, Wuhan, China; 3 School of Public Health, Tongji Medical College, Huazhong University of Science and Technology, No. 13 Hangkong Road, Wuhan, 430030 China; 4 Key Laboratory of Organ Transplantation, Ministry of Education, Wuhan, China; 5 NHC Key Laboratory of Organ Transplantation, Wuhan, China; 6 Key Laboratory of Organ Transplantation, Chinese Academy of Medical Sciences, Wuhan, China

**Keywords:** Metformin, Tuberculosis, Meta-analysis

## Abstract

**Background:**

Tuberculosis (TB) remains one of the infectious diseases with a leading cause of death among adults worldwide. Metformin, a first-line medication for the treatment of type 2 diabetes, may have potential for treating TB. The aims of the present systematic review were to evaluate the impact of metformin prescription on the risk of tuberculosis diseases, the risk of latent TB infection (LTBI) and treatment outcomes of tuberculosis among patients with diabetic mellitus.

**Methods:**

Databases were searched through March 2019. Observational studies reporting the effect of metformin prescription on the risk and treatment outcomes of TB were included in the systematic review. We qualitatively analyzed results of included studies, and then pooled estimate effects with 95% confidence intervals (CIs) of different outcome using random-effect meta-analyses.

**Results:**

This systematic review included 6980 cases from 12 observational studies. The meta-analysis suggested that metformin prescription could decrease the risk of TB among diabetics (pooled odds ratio [OR], 0.38; 95%CI, 0.21 to 0.66). Metformin prescription was not related to a lower risk of LTBI (OR, 0.73; 95%CI, 0.30 to 1.79) in patients with diabetes. Metformin medication during the anti-tuberculosis treatment is significantly associated with a smaller TB mortality (OR, 0.47; 95%CI, 0.27 to 0.83), and a higher probability of sputum culture conversion at 2 months of TB disease (OR, 2.72; 95%CI, 1.11 to 6.69) among patients with diabetes. The relapse of TB was not statistically reduced by metformin prescription (OR, 0.55; 95%CI, 0.04 to 8.25) in diabetics.

**Conclusions:**

According to current observational evidence, metformin prescription significantly reduced the risk of TB in patients with diabetes mellitus. Treatment outcomes of TB disease could also be improved by the metformin medication among diabetics.

## Background

Tuberculosis (TB) remains one of the infectious diseases with a leading cause of death among adults worldwide [1]. According to a global report from WHO, an estimated 10 million people became newly sick with TB in 2017 [1, 2]. The treatment of TB mainly depends on the special anti-TB regimen involving multiple medications. Notably, the regimen is well known for a long duration and inevitable drug side effects, which results in poor adherence to medication, and further leads to the emergence of drug resistance [3]. Given the current situation, alternative anti-TB drugs with minimal drug toxicity are needed to drive the anti-TB regimen more safe and effective.

Metformin, a first-line medication for the treatment of type 2 diabetes, showed the potential for an anti-TB treatment in recent studies [3–8]. The drug was a widely used therapy among diabetics with limited adverse events. Even a small anti-TB effect of metformin could make great sense to the treatment of TB. Metformin may shorten the standard course of anti-TB therapy and reduce the probability of drug resistance. Metformin could limit the intracellular growth of *Mycobacterium tuberculosis* (*Mtb*) through an AMPK (adenosine monophosphate-activated protein kinase)-dependent passway [4, 5]. Observational studies [9, 10] demonstrated that metformin prescription might reduce the risk of TB or latent TB infection in people with diabetes, and the studies [7, 11–13] also suggested that metformin presented an inspiring anti-TB trend on various treatment outcomes, such as increased TB treatment success, lower TB mortality, and enhanced sputum culture conversion [14]. Existing evidence seemed to sketch an antituberculotic role of metformin. However, whether metformin prescription affect the risk and treatment of TB is still nebulous.

To clarify the relationship between metformin and TB, we conducted this systematic review of observational studies (1) to summarize epidemiological evidence on the association between metformin and TB disease; (2) to figure out whether metformin prescription could reduce the risk of TB or LTBI among patients with diabetes mellitus (DM); and (3) to explore the effects of metformin prescription on the treatment outcomes of TB in diabetics.

## Methods

### Definitions

In this systematic review, latent TB infection (LTBI) represented an asymptomatic TB infection status which could be diagnosed by interferon gamma release assays. TB disease was regarded as a state of TB infection that was symptomatic and was identified in medical institutions.

### Search strategy

The protocol was registered in the PROSPERO database (CRD42019132085). We performed the research in accordance with the PRISMA (Preferred Reporting Items for Systematic Reviews and Meta-Analyses) 2009 guidelines (see Additional file 1). We identified observational studies reporting an effect of metformin prescription on the risk and treatment of TB disease. We identified eligible studies in Pubmed, Embase, and Scopus through database inception up to March 2019. Following search keywords were used to grab relevant studies: (1) tuberculosis, *Mycobacterium tuberculosis*, anti-tuberculosis, and their synonyms; (2) metformin and its other titles. The complete search strategy in details was available in the Additional file 2. The language was not limited throughout the search. References of relevant studies, if necessary, were manually checked to avoid omitting eligible studies.

### Study selection

Cohort, case-control, or cross-sectional studies exploring the impact of metformin on the risk of TB and the anti-TB therapy were included to make a full-text assessment. To fully exploit existing evidence on the association between metformin and TB, we broadened the scope of PECO (population, exposure, comparator, and outcome) framework in inclusion criteria: population was not limited; comparator could be healthy controls and patients using other antidiabetic drugs; exposure was restricted as metformin prescription; outcome could be a new diagnosis of TB disease in diabetics or indicators for the treatment of TB among patients. The main endpoints for this research were the TB-oriented composite endpoint (a new diagnosis of active TB or LTBI) and the patient-oriented composite endpoint of treatment outcomes (TB mortality, sputum culture conversion, relapse rate, and pulmonary cavities). In line with endpoints, two groups of people were included: if studies examined the risk of TB disease, their population should be diabetics without TB originally; if studies analyzed the treatment indicators, the population was patients with both DM and TB disease. We did not set any specific exclusion criteria in the selection procedure.

### Data extraction

We extracted the following data from eligible studies: (1) authors, (2) published year, (3) study location, (4) study design, (5) definite description and number of participants, (6) definition of exposure, (7) definition of outcomes, (8) definition of control group, (9) exact number of cases, (10) relevant findings, and (11) covariates adjusted in data processing.

Two investigators (XY and LL) reviewed the full-length article and extracted data from retrieved studies independently. Discrepancies in data extraction were discussed throughout the process until authors reached a mutual consensus. If necessary, we tried to contact the corresponding author of relevant articles for more information.

### Quality appraisal

Considering that researches were all observational, we used the Newcastle-Ottawa Scale (NOS) to assess the methodological quality of studies. In this research, we prepared three individual scales for cohort studies, case-control studies, and cross-sectional studies respectively. The scale for the cross-sectional study was a form specially modified for the research, and it was originally used by Fralick and colleagues [15]. Overall, quality appraisal covers three individual perspectives (the population selection; the comparability in study design; and the ascertainment of outcome or exposure). Two reviewers (XF and FC) performed the assessment separately and a senior reviewer (SC) resolved disagreements.

### Data management and statistical analyses

Our primary goal was to summarize existing epidemiological evidence of the impact of metformin on TB disease. Thus, characteristics, study framework, and findings of eligible studies were markedly collected and qualitatively analyzed. On the basis of qualitative material, we tried to pool estimate effects in meta-analyses from two perspectives: one kind reported a risk of TB in patients with metformin usage versus no metformin usage, and the other analyzed effects of metformin on varied TB treatment outcomes, such as increased TB treatment success, lower TB mortality, and enhanced sputum culture conversion.

In the analysis of onset risk, we inspected the data source along with the study period, and further selected partial researches in meta-analysis to avoid duplication of crowd data, producing a high-grade and credible result. Considering the impacts of metformin on the anti-TB treatment are diverse, we evaluated the findings of treatment outcomes separately. On the grounds that the therapeutic effect of drugs on a definite disease could only be confirmed by strictly designed clinical trials, we had not conducted an indirect comparison between different types of treatment outcomes, which was unnecessary and inefficient. Instead, we depicted the study framework and outcomes in a well-designed table to display the current status on this topic, and we merged the same type of results to generate pooled results.

We conducted random-effects meta-analyses to combine the estimates from selected researches reported as hazard ratios (HRs), odds ratios (ORs) and relative risks (RRs). Since TB incidence was low in studies, we regarded RRs as similar approximations of OR and merged them with HRs, resulting in a common estimate effect of OR [16]. If an article included more than one study, we deconstructed the studies separately. Due to the limited studies in a single meta-analysis, sensitivity analysis and publication bias assessment were not planned. Heterogeneity of the study-specific estimates was assessed with the Cochran’s Q test and *I*^*2*^ statistics (0–100%), and I^2^ < 50% was regarded as low heterogeneity, I^2^ value 50 to 75% as moderate heterogeneity, I^2^ > 75% as substantially high heterogeneity [17]. All *P*-values were two-tailed and *P* < 0.05 was used to define the significant level. Statistical analyses in this research were performed on Stata version 14.0.

## Results

### Study characteristics and quality appraisal

**Fig. 1:**
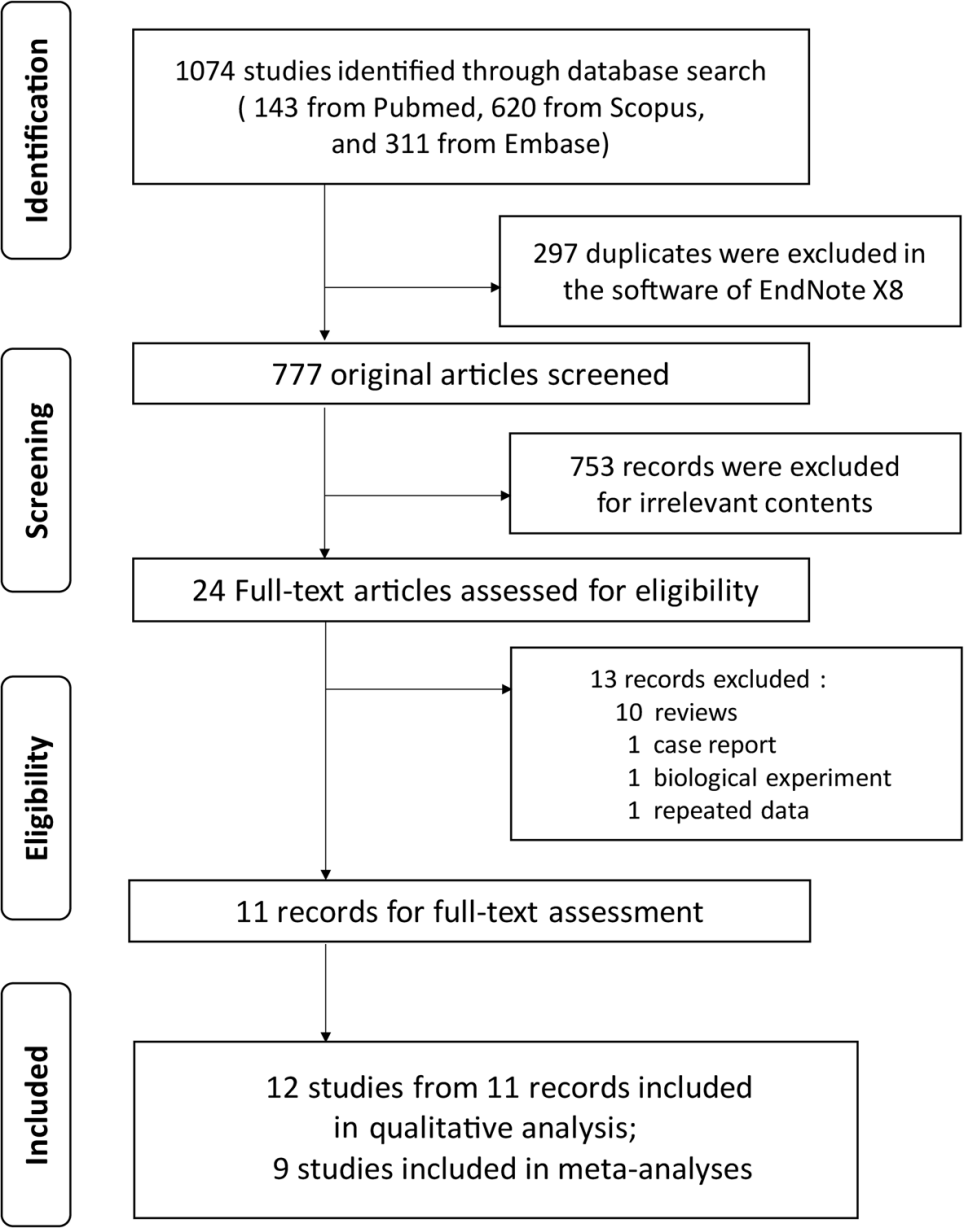
Study screening flowchart

The literature searching strategy of this systematic review was displayed in Fig. 1. A pilot research [4] and a cross-sectional study [18] used the same data set, and we chose the former to analyze the overlapped data. Thus, eleven individual articles [4, 7–13, 19–21] with 12 studies satisfying inclusion criteria were identified in the systematic review, and study characteristics were reported in Table 1. Cases, controls and outcomes of each study were carefully extracted and inspected. Of 12 eligible studies, one was a case-control design study, one was of cross-sectional design, and the rest were retrospective cohort studies. Sample sizes ranged from 58 to 177,732. Six studies were from Taiwan, and one from U.S.A., India, South Korea, Singapore, China (mainland) respectively. The participants were patients with DM for 8 studies investigating the risk of TB or LTBI, and diabetics with TB for 4 studies discussing the treatment outcomes of TB disease. The outcomes for TB treatment were as follows: (1) sputum culture conversion, (2) relapse of TB, (3) TB mortality, (4) success treatment, and (5) number of pulmonary cavities. The methodological quality of included studies was fully assessed and manifested in Table 2. All studies had moderate to high quality (NOS quality score ≥ 6). To better present study framework, we summarized basic characteristics in Table 3. Overall, ten studies showed a statistical significant anti-TB effect of metformin, while the other two were the opposite.

**Table 1:** Characteristics of included studies in the systematic review

Study Authors and Published Year (location)	Study Design	Participants	Exposure	Outcomes	Controls	Case	Result	Adjustment of covariates
Magee et al. 2018 [8] (U.S.A.)	Cross-sectional study	Patients with diabetes identified by self-report and glycated hemoglobin (*n* = 4958)	Metformin medication identified by self-report	**A diagnosis of LTBI infection** identified by QuantiFERON-TB Gold In-tube	Patients without metformin medication	575	Latent TB infection prevalence was non-significantly higher in those without metformin use (prevalence difference, 1.4, 95%CI, −3.7 −6.4%) compared to those self-reporting any metformin use (OR, 1.1, 95%CI, 0.7 to 1.9).	NA
Singhal et al. 2014 [4] (Singapore)	Two retrospective cohort studies	**Cohort 1:** Patients suffered from both DM and TB (*n* = 273) **Cohort 2:** Patients with DM (*n* = 220)	Metformin treatment	**Cohort 1: Pulmonary cavities** at diagnosis and **mortality** during the first year after diagnosis. **Cohort 2:** A **diagnosis of LTBI** tested by T-Spot TB array	Patients using alternative drugs for DM.	**Cohort 1:** 273 **Cohort 2:**62	**Cohort 1:** those receiving metformin had fewer pulmonary cavities (OR, 0.6; 95%CI, 0.36 to 0.97). The Mortality was 3% among patients who received metformin compared to 10% among patients in the non-metformin group (OR, 0.29; 95%CI, 0.14 to 0.95). **Cohort 2:** metformin therapy was associated with reduced T-SPOT reactivity when compared with controls (OR, 0.44; 95%CI, 0.20 to 0.95).	NA
Marupuru et al. 2017 [19] (India)	Case-control study	Diabetics (≥ 40 years old) identified on the basis of ICD-10 coding for disease classification (E11.9, n = 448)	Metformin usage	The **diagnosis of TB** following ICD-10 code A15-A19	Patients without metformin usage	149	The protective effect of metformin against TB was 3.9-fold in diabetics (OR = 0.256, 0.16 to 0.40). No difference was found between those on 1000 mg metformin (27.3%), and on 500 mg (25.7%) for development of TB.	NA
Lee et al. 2017 [11] (South Korea)	Retrospective cohort study	Culture-proven pulmonary TB in patients diagnosed with DM; follow-up sputum *Mtb* cultures after 2 months of treatment; completion of World Health Organization-recommended TB treatment (*n* = 105)	Metformin treatment	(1) **Sputum culture conversion** after 2 months of treatment; (2) **Recurrence** of TB proven by isolation of *Mtb* or clinical radiological evidence	Participants without metformin treatment	105	The OR of sputum culture conversion at 2 months for patients with metformin use was 2.69 (95%CI, 0.92 to 7.95); A statistical difference in the recurrence rate with the use of metformin (OR, 1.92; 95%CI, 0.42 to 8.76) was observed. Metformin improved the sputum culture conversion rate in patients with cavitary pulmonary TB (OR, 10.8; 95%CI, 1.22 to 95.63)	Sex, statin use, insulin, cancer, AFB smear grade, and drug resistance
Lin et al. 2017 [20] (Tai Wan)	Retrospective cohort study	Patients aged 20 and older newly diagnosed with diabetes (ICD-9250.XX and A-code A181) and without a past history of pulmonary TB (*n* = 22,256)	Metformin treatment	**Incidence** of pulmonary TB identified in follow-up	Patients without metformin treatment	682	Unadjusted crude HR: 0.42 (95%CI, 0.35 to 0.50) Adjusted HR: 0.52 (95%CI, 0.43 to 0.62)	Sex, age, alcoholism, chronic obstructive pulmonary disease, cirrhosis, alcoholic liver damage, hepatitis C, chronic kidney diseases, and malignancies
Degner et al. 2018 [12] (Tai Wan)	Retrospective cohort study	Patients aged ≥13 years with culture-confirmed, drug-susceptible pulmonary TB undergoing treatment and diabetes (*n* = 634)	Metformin medication within 30 days of starting TB treatment	Effect on **mortality** among patients with DM undergoing TB treatment	Patients without metformin prescriptions	634	Adjusted HR: 0.56 (95%CI, 0.39 to 0.82) Unadjusted HR: 0.50 (95%CI, 0.35 to 0.72)	Age, sex, chronic kidney disease, cancer, cavitary diseases, TB treatment adherence
Lee et al. 2018 [9] (Tai Wan)	Retrospective cohort study	Patients had at least one hospital admission or at least three outpatient visits with a DM diagnostic code (ICD-9) within 365 calendar days (*n* = 177,732)	With total prescriptions of metformin for *>* 90 cumulative defined daily doses within 1 year after the onset of DM treatment	A **newly diagnosed TB** (ICD-9-CM code: 010–018) after the index date.	Diabetics do not satisfy the exposure criteria	1514	Adjusted HR: 0.84 (95%CI, 0.74 to 0.96) TB risk was lower in high-dose metformin users than in low-dose users (HR, 0.83; 95%CI, 0.72 to 0.97)	Sex, type 1 diabetes mellitus, age, low income, chronic obstructive pulmonary disease, liver cirrhosis, pulmonary cancer, extra-pulmonary cancer, bronchiectasis, and so on
Lin et al. 2018 [10] (Tai Wan)	Retrospective cohort study	Patients who were 20–100 years old and who were newly diagnosed with type 2 DM (ICD-9-CM, 250.X0 and 250.X2) (*n* = 10,052)	Metformin usage	A **diagnosis of active TB** (ICD-9-CM, 010–018) during the follow-up period and the prescription of more than two anti-TB medications for more than 90 days.	Participants without metformin usage	329	Adjusted RR: 0.24 (95%CI, 0.18 to 0.32) Unadjusted RR: 0.37 (95%CI, 0.29 to 0.47)	The duration of DM diagnosis, comorbidities (chronic pulmonary disease/ renal disease), oral anti-diabetic therapy, and insulin injection therapy
Ma et al. 2018 [21] (China)	Retrospective cohort study	Culture-positive retreatment pulmonary TB patients with type 2 DM Multidrug-resistant TB, extensively drug-resistant TB, and extra-pulmonary TB were excluded.(*n* = 58)	Metformin treatment in regimens for diabetes	**(1) Success treatment of TB** **(2) Sputum culture conversion** by the end of 2 months **(3) Relapse rates** of patients	Patients without metformin medication	58	There were a higher proportion of treatment success (OR, 6.00; 95%CI, 0.71 to 50.59) and sputum culture conversions by the end of 2 months (OR, 2.80; 95%CI, 0.55 to 14.23) among metformin group. The relapse rates of patients in the metformin and non-metformin group were 6.3 and 35.7% (OR, 0.12; 95%CI, 0.01 to 1.20).	NA
Pan et al. 2018 [13] (Taiwan)	Retrospective cohort study	Patients with a diagnosis of type 2 DM (ICD-9250 × 0, 250 × 2). Patients aged < 20 years or had a diagnosis of TB were excluded.(*n* = 9475)	Participants received ≥60 cumulative defined daily dose of metformin and < 15 cumulative defined daily dose of sulfonylurea	**TB occurrence** (ICD-9, 010–018)	Participants received ≥60 cumulative defined daily dose of sulfonylurea and < 15 cumulative defined daily dose of metformin	263	Adjusted RR: 0.337 (95%CI, 0.169 to 0.673) Unadjusted RR: 0.477 (95%CI, 0.268 to 0.850)	Age, sex, adapted diabetes complication severity index score, index year, income level, and comorbidities
Tseng et al. 2018 [7] (Taiwan)	Retrospective cohort study	Newly diagnosed diabetes patients (ICD-9) who had been followed up in the outpatient clinics with a prescription of antidiabetic drugs for two or more times (*n* = 164,267)	Patients had been prescribed metformin as the first antidiabetic drug	**Incidence density** of TB infection	Patients without metformin prescriptions	2336	Adjusted HR: 0.552 (95%CI, 0.493–0.617) Unadjusted HR: NA	Age, diabetes duration, sex, occupation, living region, hypertension, dyslipidemia, obesity, diabetes-related complications, antidiabetic drugs and so on

Abbreviations: *TB* Tuberculosis, *LTBI* Latent tuberculosis infection, *DM* Diabetes mellitus, *Mtb Mycobacterium tuberculosis*, *HR* Hazard ratio, *OR* Odds ratio, *RR* Relative ratio, *CI* Confidence interval, *ICD* International classification of diseases, *AFB* Acid-fast bacilli, *NA* Not available

**Table 2:** Newcastle-Ottawa scale for assessing the quality of included studies

Study design	Author, year	Selection (Max = 4)	Comparability (Max = 2)	Outcome (Max = 3)	Overall quality score (Max = 9)
Cross-sectional study	Magee et al. 2018 [8]	3	2	3	8
Study design	Author, year	Selection (Max = 4)	Comparability (Max = 2)	Exposure (Max = 3)	Overall quality score (Max = 9)
Case-control study	Marupuru et al. 2017 [19]	4	1	2	7
Study design	Author, year	Selection (Max = 4)	Comparability (Max = 2)	Outcome (Max = 3)	Overall quality score (Max = 9)
Cohort study	Tseng 2018 [7]	4	2	2	8
	Pan et al. 2018 [13]	4	2	2	8
	Ma et al. 2018 [21]	4	1	3	8
	Lin et al. 2018 [10]	4	2	2	8
	Lee et al. 2018 [9]	4	2	2	8
	Degner et al. 2018 [12]	4	2	2	8
	Lin et al. 2017 [20]	4	2	2	8
	Lee et al. 2017 [11]	4	2	2	8
	Singhal et al. 2014 [4]	3	2	1	6

**Table 3:** Study framework of included studies

Author Year	Pubmed ID	Study period	Data source, country or region	Study design	Population	Outcome	Does metformin show a significant anti-tuberculosis effect?	Risk of bias assessment (study quality)
Marupuru et al. 2017 [19]	28,199,824	2011–2015	Kasturba Hospital, India	Case-control	Patients with DM (*n* = 448)	A diagnosis of TB	Yes	7
Lin et al. 2017 [20]	NA	2000–2006	NHIRD (database), Taiwan	Cohort	Patients with DM (n = 22,256)	A new diagnosis of TB	Yes	8
Lee et al. 2018 [9]	30,335,800	2003–2006	NHIRD (database), Taiwan	Cohort	Patients with DM (n = 177,732)	A new diagnosis of TB	Yes	8
Lin et al. 2018 [10]	29,943,489	1998–2010	NHIRD (database), Taiwan	Cohort	Patients with type 2 DM (n = 10,052)	A new diagnosis of TB	Yes	8
Pan et al. 2018 [13]	29,253,553	2003–2013	NHIRD (database), Taiwan	Cohort	Patients with type 2 DM (n = 9475)	A new diagnosis of TB	Yes	8
Tseng et al. 2018 [7]	30,205,606	1999–2005	NHIRD (database), Taiwan	Cohort	Patients with type 2 DM (n = 164,267)	A new diagnosis of TB	Yes	8
Lee et al. 2017 [11]	29,540,054	2011–2012	Seoul national university hospital, South Korea	Cohort	Patients with both DM and pulmonary TB (n = 105)	(1) Sputum culture conversion; (2) Recurrence of TB	(1) No (2) No	8
Ma et al. 2018 [21]	29,679,254	2009–2013	Five hospitals in China, China	Cohort	Patients with both type 2 DM and pulmonary TB (n = 58)	(1) Sputum culture conversion; (2) Recurrence of TB (3) Success treatment	(1) No (2) No (3) No	8
Degner et al. 2018 [12]	29,325,084	2000–2013	NTUH (hospital) Taiwan	Cohort	Patients with both DM and pulmonary TB (n = 634)	TB mortality	Yes	8
Magee et al. 2018 [8]	30,523,163	2011–2012	NHANES (database) U.S.A.	Cross-sectional	Patients with DM (n = 4958)	A diagnosis of LTBI by QFT	No	8
Singhal et al. 2014 [4]	25,411,472	NA	Tan Tock Seng hospital, Singapore	Cohort	**Cohort 1**: Patients with both DM and pulmonary TB (n = 273); **Cohort 2**: Patients with DM. (n = 220)	(1) TB mortality (2) Number of pulmonary cavities (3) A diagnosis of LTBI by T-spot	(1) Yes (2) Yes (3) Yes	6

Abbreviations: *TB* Tuberculosis, *LTBI* Latent tuberculosis infection, *DM* Diabetes mellitus, *QFT* QuantiFERON-TB Gold In-tube, *NHIRD* National Health Insurance Research Database, *NTUH* National Taiwan University hospital, *NA* Not available

### Metformin prescription and the risk of TB disease

**Fig. 2:**
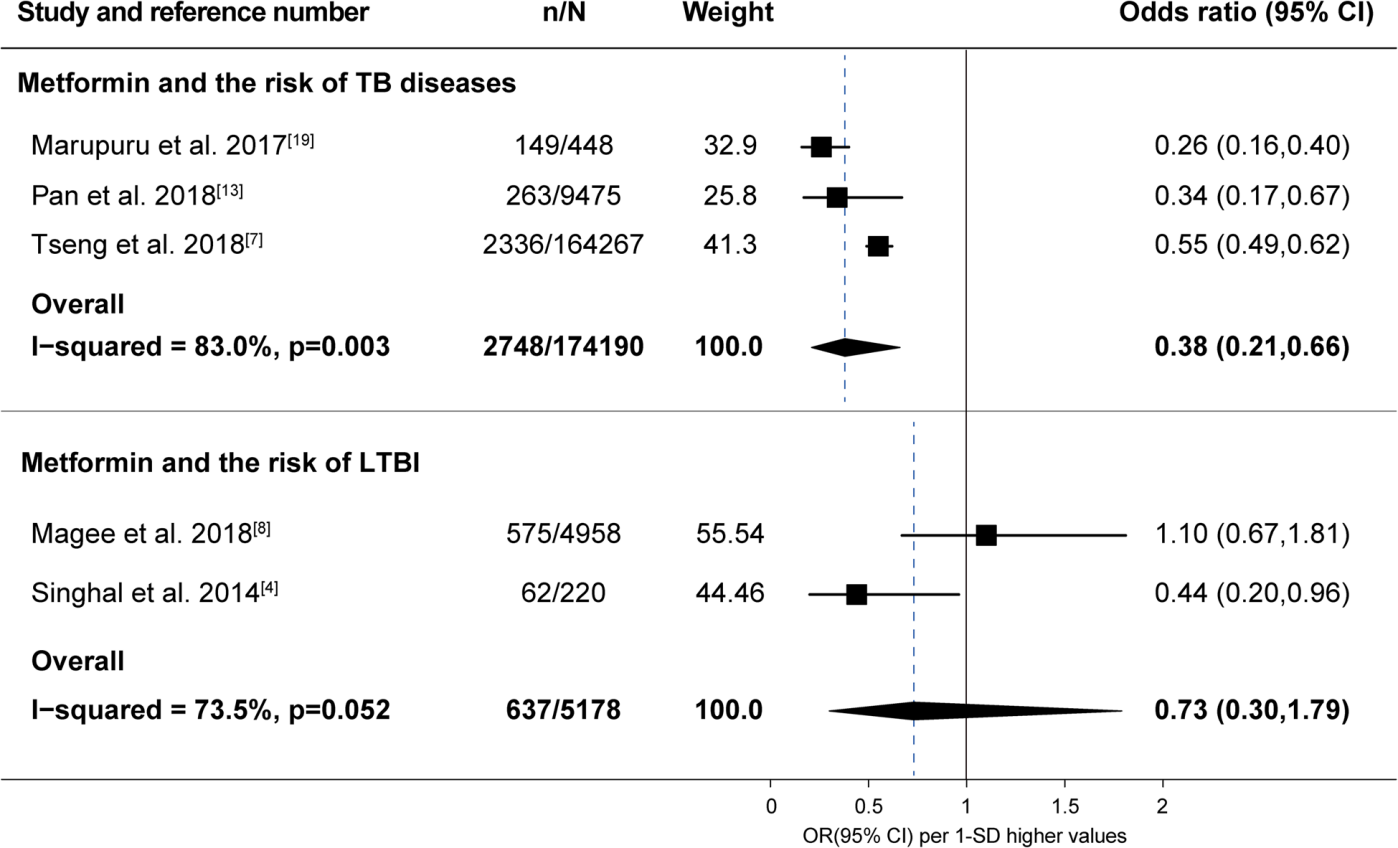
Forest plot of the association between metformin and the risk of TB disease or LTBI. Abbreviations: TB, tuberculosis; LTBI, latent tuberculosis infection; n, number of cases; N, number of participants; CI, confidence interval; OR, odds ratio; SD, standard deviation

Six studies [7, 9, 10, 13, 19, 20] carrying 5273 cases advocated a reduced risk of TB among metformin users compared to non-users, five of which were performed using the same database (National Health Insurance Research Database from Taiwan). To avoid repeated analysis of crowd data, we only included two Taiwan studies [7, 13] using that database. Eventually, three studies (two studies in Taiwan and one study in India) were included in the meta-analysis (Fig. 2) considering sample sizes and study population. Despite substantial heterogeneity between studies, the pooled analysis (see Fig. 2) and the six individuals suggested metformin usage could decrease the risk of TB among diabetics (OR, 0.38; 95%CI, 0.21 to 0.66). However, the meta-analysis (see Fig. 2) exploring the potential risk of LTBI in patients with DM reported that metformin prescription was not statistically related with the onset of LTBI (OR, 0.73; 95%CI, 0.30 to 1.79).

### The impact of metformin on treatment outcomes of TB disease

**Fig. 3:**
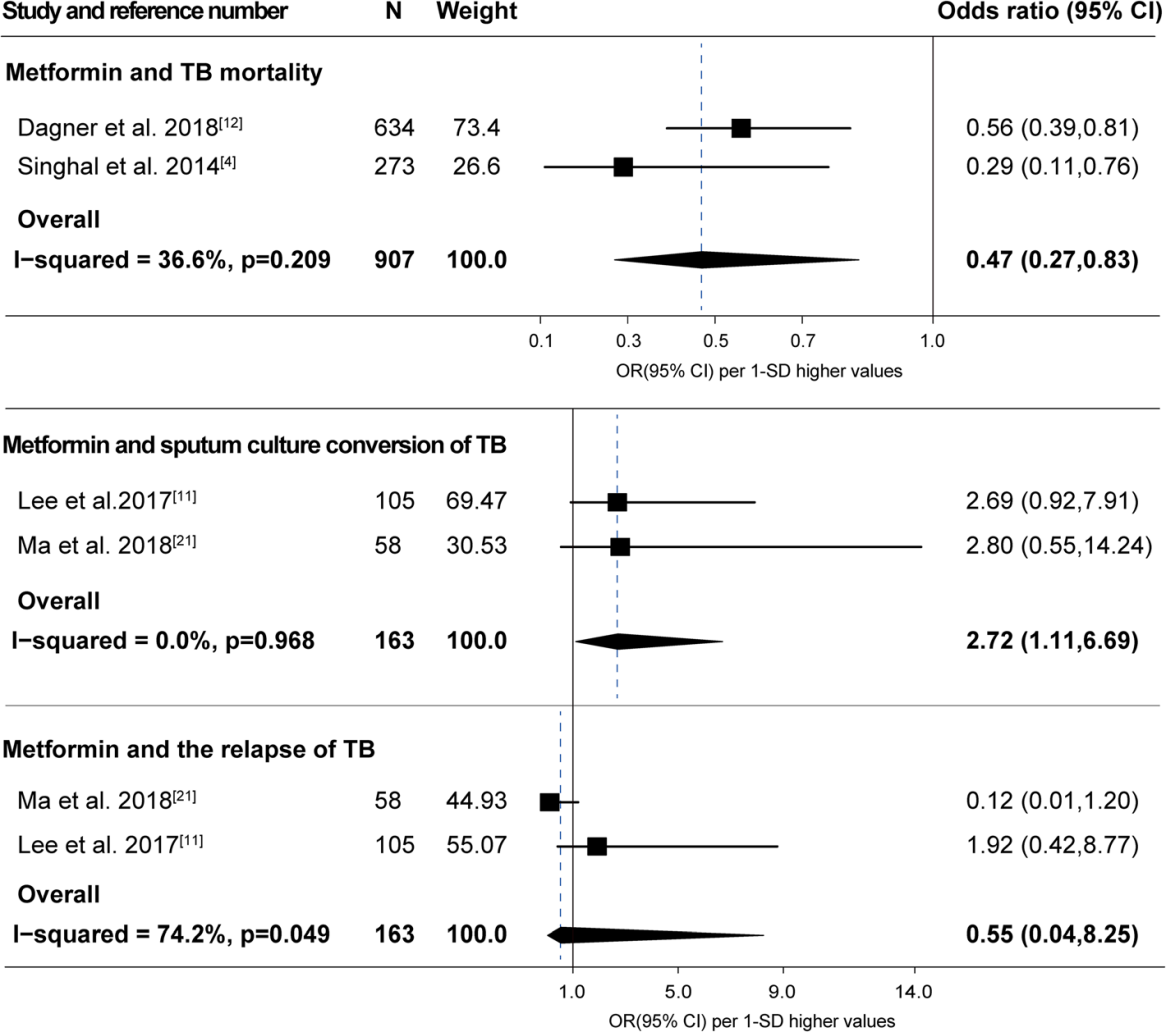
Forest plot of the association between metformin and treatment outcomes for TB disease. Abbreviations: TB, tuberculosis; N, number of participants; CI, confidence interval; OR, odds ratio; SD, standard deviation

According to the combined analyses (see Fig. 3), the TB mortality could be limited by metformin usage (OR, 0.47; 95%CI, 0.27 to 0.83), and the sputum culture conversion at 2 months of TB disease was significantly promoted in metformin users compared to non-users (OR, 2.72; 95%CI, 1.11 to 6.69). Contrarily, the pooled result of the relapse of TB within 3 years was not statistically different (OR, 0.55; 95%CI, 0.04 to 8.25) between the metformin group and the control group.

## Discussion

In this comprehensive systematic review from 12 observational studies among diabetics, metformin prescription is significantly associated with a decreased risk of TB disease, a smaller TB mortality, and a higher probability of sputum culture conversion at 2 months. Metformin prescription could not reduce the risk of LTBI and the relapse rate of TB disease. The result supports an auxiliary anti-tuberculosis role of metformin in patients with DM.

Due to the different study design, study population and the definition of outcomes (see Table 3), the meta-analysis on the risk of TB showed substantial heterogeneity (see Fig. 2, *I*^*2*^ = 83.0%, *P* = 0.003). The diagnostic methods of LTBI (see Table 3) in the study by Singhal et al. [4] (T-SPOT.TB) and Magee et al. [8] (QFT, QuantiFERON-TB Gold In-tube) were both based on T-cell interferon-gamma release assays, however, in distinct approaches. The difference in diagnosis method may lead to obvious between-study heterogeneity (see Fig. 2, *I*^*2*^ = 73.5%, *P* = 0.052). The analysis of the impact of metformin on the relapse of TB also showed obvious heterogeneity (see Fig. 3, *I*^*2*^ = 74.2%, *P* = 0.049).

Individual studies [7, 9, 10, 13, 19, 20] and pooled analysis provide sufficient evidence that patients with DM initiating metformin usage have a lower risk of TB disease compared to non-users. However, this phenomenon does not occur in the detection of LTBI. TB is known as a granulomatous inflammatory disease involving a special immunopathology. Most TB infections do not induce symptoms among patients, which is regarded as LTBI, and only a part of them turn into active TB disease in those who have a weakened immune system [22]. Accordingly, metformin could enhance *Mtb*-specific host immunity [4]. Combining the epidemiological evidence, we speculate that metformin treatment could inhibit the conversion of LTBI to active TB disease. This finding is consistent with previous studies [4, 5, 14] that metformin could only function as a host-directed therapy rather than an *Mtb-* directed therapy.

Metformin treatment in the course of anti-TB regimen could improve treatment outcomes among patients with both DM and TB. However, metformin prescription could not limit the relapse of TB disease. Recent studies [4, 14, 21] indicate that the anti-tuberculosis effect of metformin is dependent on the standard regimen for TB, and metformin only acts as a supporting role with long-term medication. Two studies [11, 21] suggest that metformin usage could not reduce the sputum culture conversion in patients with DM. We consider the small sample size substantially reduce the statistical power, and the deficiency has been solved by the meta-analysis (see Fig. 3). The impact of metformin on the treatment of TB is inspiring. However, to ensure the therapeutic effect of metformin on TB, well-designed randomized controlled trials are necessary.

The mechanism of how metformin affects the human immune system to *Mtb* has been explored in biological experiments [4, 14]. Metformin affects immune response and inflammation in multiple approaches. The protecting effect of metformin on TB patients is mediated by increased host cell production of mitochondrial reactive oxygen species and increased acidification of mycobacterial phagosome [4]. Metformin treatment also shows an anti-inflammatory effect through the activation of AMPK, a negative regulator of inflammation process [4]. At the individual level, metformin administered to healthy human beings leads to significant down-regulation of genes functioned in oxidative phosphorylation, mammalian target of rapamycin (mTOR) signaling and type I interferon response pathways, following stimulation with *Mtb* [14]. Experimental evidence supports the host-directed effect of metformin on TB disease.

Metformin is used to prevent the onset of DM in high-risk populations [23], while DM increases the risk of TB disease and adverse TB outcomes [24]. Combined with our findings, we suggest that metformin usage may prevent DM and TB at the same time. Moreover, metformin approaches a host-directed therapy for TB, which could (1) amplify the efficacy of antibiotic-based treatment, (2) shorten anti-TB regimens, and (3) promote the development of immunological memory that reduces the relapse of TB [4].

### Strengths and limitations

The research was the first systematic review that focused on the association between metformin and TB. We tried to improve the efficiency of meta-analyses by minimizing data duplication and analyzing diverse treatment outcomes of TB. However, due to the limited data from original studies, we failed to conduct meta-regressions, dose-effect analyses and subgroup analyses. This research was based on diabetics, and whether metformin could affect the risk and treatment of TB among people without DM was still unclear. Because the study design and the population varied between included studies, there was substantial heterogeneity in meta-analyses, and the heterogeneity could not be eliminated by the subgroup analysis or sensitivity analysis for the limited data.

## Conclusion

In conclusion, this systematic review indicate that usage of metformin could significantly reduce the risk of TB disease among diabetics. Treatment outcomes of TB could be improved by metformin prescription in patients with DM. To confirm therapeutic and preventive effects of metformin on TB, well-designed randomized controlled trials, however, in patients with or without DM, are needed.

## Supplementary information

**Additional file 1.** PRISMA checklist.

**Additional file 2.** Search Strategy.

## Data Availability

All relevant data for this study are presented in tables, figures and supplementary materials.

## Abbreviations

AMPK: Adenosine monophosphate-activated protein kinase
CI: Confidence interval
DM: Diabetes mellitus
HR: Hazard ratio
LTBI: Latent tuberculosis infection
*Mtb*: *Mycobacterium tuberculosis*
mTOR: Mammalian target of rapamycin
NOS: Newcastle-Ottawa Scale
OR: Odds ratio
PECO: Population, exposure, comparator, and outcome
PRISMA: Preferred Reporting Items for Systematic Reviews and Meta-Analyses
RR: Relative risk
TB: Tuberculosis
