# Supplementary material for: Impact of metformin on the risk and treatment outcomes of tuberculosis in diabetics: a systematic review
Source: BMC Infect Dis. 2019 Oct 17;19:859. doi: 10.1186/s12879-019-4548-4 (PMC6796338; doi:10.1186/s12879-019-4548-4)
Supplement: Supplementary file 2 — Additional file 2. Search Strategy. [file 12879_2019_4548_MOESM2_ESM.docx]

**Search strategy**

1. Pubmed

(tuberculosis OR tubercle bacillus OR mycobacterium tuberculosis OR TB OR mycobacteria OR antituberculosis) AND (metformin OR glucophage OR dimethylbiguanide OR difres OR metformin hydrochloride OR carbophene OR biomet OR mortamet OR glumetza OR ouimet OR gliformin OR dianeben OR diabex OR diaformin OR siofor OR Gilford)

1. Scopus

(TITLE-ABS-KEY (tuberculosis OR mycobacterium OR tb OR mycobacteria OR antituberculosis)) AND (TITLE-ABS-KEY (metformin OR glucophage OR dimethylbiguanide OR difres OR hydrochloride OR carbophene OR biomet OR mortamet OR glumetza OR ouimet OR gliformin OR dianeben OR diabex OR diaformin OR siofor OR gilford))

1. Embase

((tuberculosis or tubercle bacillus or mycobacterium tuberculosis or TB or mycobacteria) and (metformin or glucophage or dimethylbiguanide or difres or metformin hydrochloride or carbophene or biomet or mortamet or glumetza or ouimet or gliformin or dianeben or diabex or diaformin or siofor or gilford)).af.
